# A cohort study of adolescents with depression in China: tracking multidimensional outcomes and early biomarkers for intervention

**DOI:** 10.1136/gpsych-2022-100782

**Published:** 2022-08-26

**Authors:** Xiaofei Zhang, Yanling Zhou, Jiaqi Sun, Ruilan Yang, Jianshan Chen, Xiaofang Cheng, Zezhi Li, Xinlei Chen, Chanjuan Yang, Xinhong Zhu, Liping Cao

**Affiliations:** 1 Department of Child and Adolescent Psychiatry, Early Detection and Intervention Center for Adolescent Mood Disorders, Affiliated Brain Hospital of Guangzhou Medical University, Guangzhou, Guangdong, China; 2 Department of Radiology, Affiliated Brain Hospital of Guangzhou Medical University, Guangzhou, Guangdong, China; 3 Deparment of Adult Psychiatry, Affiliated Brain Hospital of Guangzhou Medical University, Guangzhou, Guangdong, China; 4 Brain Disease and Health Reaserch Center, Pazhou Laboratory, Guangzhou, Guangdong, China

**Keywords:** depression, adolescent psychiatry, genetics, behavioural, biological psychiatry, cohort studies

## Abstract

**Background:**

Depression in adolescents is recognised as a global public health concern, but little is known about the trajectory of its clinical symptoms and pathogenesis. Understanding the nature of adolescents with depression and identifying early biomarkers can facilitate personalised intervention and reduce disease burden.

**Aims:**

To track multidimensional outcomes of adolescents with depression and develop objective biomarkers for diagnosis, as well as response to treatment, prognosis and guidance for early identification and intervention.

**Methods:**

This is a multidimensional cohort study on the Symptomatic trajectory and Biomarkers of Early Adolescent Depression (sBEAD). We planned to recruit more than 1000 adolescents with depression and 300 healthy controls within 5 years. Multidimensional clinical presentations and objective indicators are collected at baseline, weeks 4, 8, 12 and 24, and years 1, 2, 3, 4 and 5.

**Conclusions:**

To the best of our knowledge, this is the first longitudinal cohort study that examines multidimensional clinical manifestations and multilevel objective markers in Chinese adolescents with depression. This study aims at providing early individualised interventions for young, depressed patients to reduce the burden of disease.

**Trial registration number:**

Chinese Clinical Trial Registry ID ChiCTR2100049066.

**What is already known on this topic**

- Depression in adolescents is a heterogeneous and complicated disease with a lack of objective biomarkers.

**What this study adds**

- We will identify distinct subtypes of adolescent depression by tracking the trajectory of clinical symptoms, diagnosis, and functional outcomes over a five-year follow-up period, ultimately creating highly homogeneous cohorts.

**How this study might affect research, practice or policy**

- Our multidimensional time-dependent database will provide vital clues to identify the objective markers, which will ultimately promote the development of neurobiological-based diagnostic classification and early individualized interventions for adolescents with depression.

## Introduction

Depression is a global public health problem that affects all ages.^1^ In recent years, the prevalence of depression in adolescents has exceeded that of adults.^2^ In the USA, the lifetime prevalence of a major depressive disorder (MDD) in adolescents is 11.0%, with a 12-month prevalence of 7.5%.^3^ According to the latest epidemiological data in China, the pointed prevalence of MDD in adolescents is 2%, higher than in previous surveys.^4^ Research suggests that depression in adolescents is associated with high recurrence rates, premature death, poor academic outcomes and psychosocial dysfunction, which may persist into adulthood or lead to other mental disorders,^5 6^ emphasising the need for early identification and intervention.

The biggest obstacle to early identification and intervention is the lack of objective markers of illness risk, progression, or response to treatment, although previous studies have explored biomarkers in genetics, neurobiology and gut flora, and so on.^7 8^ Lack of biomarkers leads to low treatment rates^9^ and high rates of misdiagnosis,^1^ especially in differentiating bipolar disorder from unipolar depression.^10^ The failure to resolve these issues, as well as the lack of combined multilevel units of biomarkers, may be due to the complexity and developmental heterogeneity of adolescents with depression. According to previous studies, one of the main sources of heterogeneity is clinical manifestations. The clinical characteristics of depression in adolescents differ from those of adults, and they manifest in more self-injury, suicidal thoughts and behaviours, comorbidity with other mental disorders and physical problems, sleep rhythm disturbances, and use of tobacco, alcohol or other substances.^7^ Another issue is the different patterns of disease persistence and clinical outcomes in adolescents with depression. About 30%–55% of individuals experiencing a depressive episode have subthreshold manic symptoms,^11 12^ while only 13%–33% of children and adolescents with depressive disorder may develop bipolar disorder.^10 13^ Thus, the age-dependent heterogeneity of depressive symptoms should be considered. In addition, adopting dimensional conceptualisations may help us understand the full spectrum of depression in adolescents better. Therefore, dividing the clinical presentation of research subjects into different dimensions based on the development of characteristics of symptoms, risk factors, and functional outcomes may provide important information regarding the ways by which psychopathology gradually emerges throughout development. Moreover, the combination of different biomarkers cutting across different levels of disease complexity may lead to the identification of objective biomarkers for adolescents with depression.^14^ Therefore, it is essential to conduct long-term longitudinal observation to capture the biopsychosocial profile for identifying accurate biomarkers.

**Figure 1:**
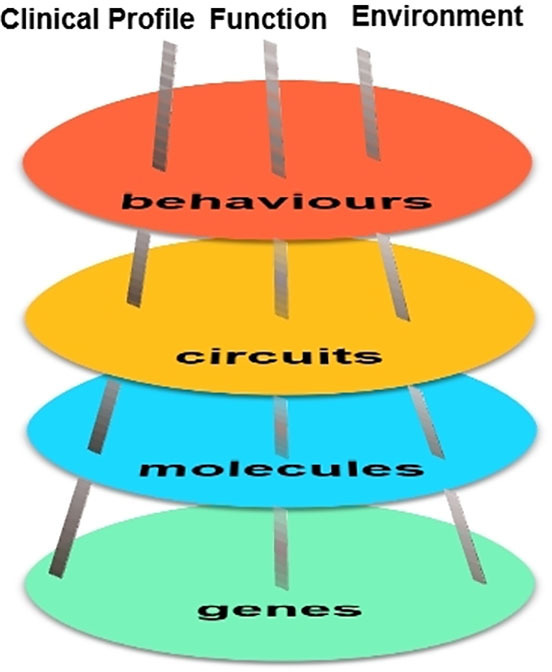
Comprehensive framework of multidomain and multilevel units in our cohorts. The multidomain in this cohort included clinical profile, functional outcome, environmental risk factors and biological information. Multilevel units in this cohort stands for different levels of biological information, including genes, molecules, circuits and behaviours.

At present, there have been extensive cohort studies performed abroad; however, most of the world-leading cohorts are birth cohort studies.^5 15^ There are a few cohort studies of adolescents with depression, with sample sizes ranging from 200 to 500 and follow-up periods of up to 5 years,^16 17^ but overall there is a lack of large-scale cohort studies on adolescents with depression, especially those with a multidimensional comprehensive framework design.^18^ There is no previous cohort study specifically of adolescents with depression in China. To address this gap, our team has embarked on a cohort study on the Symptomatic trajectory and Biomarkers of Early Adolescent Depression. Given the main deficiencies in the current process of searching for biomarkers of depression, we designed a comprehensive framework of multidomain and multilevel units (figure 1) and facilitated the identification of biomarkers reflecting relationships among genetic, molecular neural circuitry levels and behavioural level abnormalities, all pointing to the development of early interventions for adolescents with depression and reducing the burden of disease.

## Objective

### Main objective

The main purpose of this comprehensive longitudinal framework study is to develop objective markers for the diagnosis, disease progression and response to treatment, guiding early identification and intervention for adolescent depression.

### Secondary objectives

1. We are tracking the trajectory of clinical symptoms, diagnosis and functional outcomes over a prolonged follow-up period, providing detailed information about time-dependent heterogeneity of clinical symptoms in Chinese adolescents with depression.
2. Furthermore, we will use comprehensive longitudinal data to perform neurobiologically-based diagnostic classification, independent of disorder-based categories.

## Methods

### Study design and participants

This multidimensional cohort study of adolescents with depression began in July 2021 and will end in June 2026. It is being carried out at the Affiliated Brain Hospital of Guangzhou Medical University, Guangzhou, Guangdong Province, China.

Considering that the average time from the first depressive episode to mania in depressed patients is 2–4 years, and the conversion rate from depression to bipolar disorder is highest in the first 5 years,^19^ we planned a 5-year follow-up period. The first available face-to-face clinical interview was taken at the baseline time point (T1) for each participant. After that, participants were/will be followed up separately at T2 (week 4), T3 (week 8), T4 (week 12), T5 (week 24), T6 (year 1), T7 (year 2), T8 (year 3), T9 (year 4) and T10 (year 5). We planned to recruit more than 1000 adolescents with depression and 300 healthy controls from July 2021 to June 2026. Of note, we are using the Bipolar Prodrome Symptom Scale-Full Prospective (BPSS-FP) to assess bipolar high-risk syndrome for individuals who experience subthreshold manic symptoms.^20^ When a patient meets the bipolar high-risk syndrome threshold, they are enrolled in a bipolar disorder high-risk subcohort and are followed for possible progression into bipolar disorder. In addition to the regular follow-up time points mentioned above, this subcohort have unplanned visits during possible hypomanic episodes.

**Figure 2:**
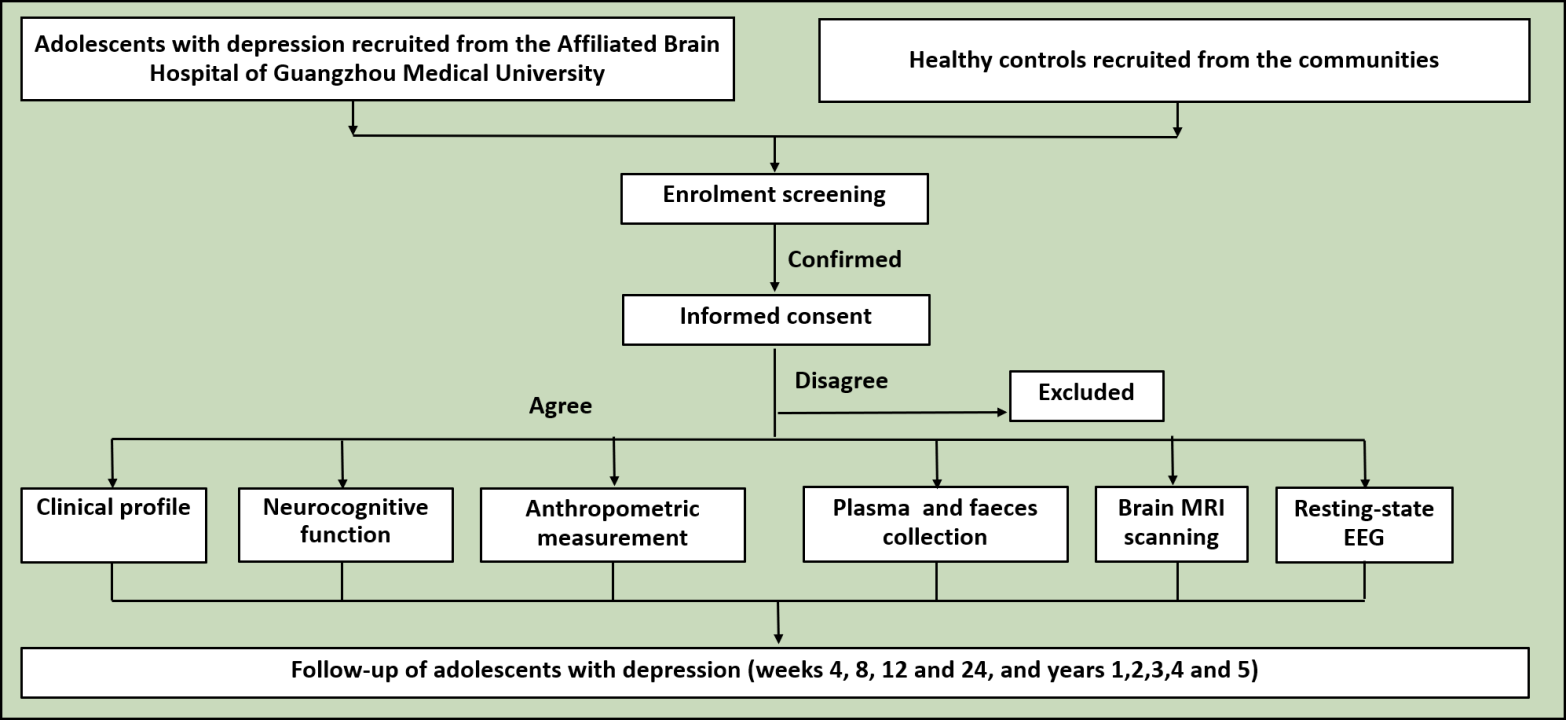
Flowchart of the multidimensional cohort study of adolescents with depression.

The study flowchart is presented in figure 2.

### Participant eligibility criteria

The inclusion criteria for patients are as follows:

1. Meet the diagnostic criteria for MDD according to the Diagnostic and Statistical Manual of Mental Disorders, Fifth Edition (DSM-5).^21^
2. Drug-naive or continuous psychiatric pharmacotherapy for less than 14 days.
3. Aged 9–18 years.
4. Voluntary participation with written informed consent from the legal guardian.

The exclusion criteria for patients are as follows:

1. Neurological diseases, mental disorders due to brain diseases and physical diseases.
2. Developmental disorders, except for attention-deficit hyperactivity disorder, including IQ ≤70 or mental retardation, autistic spectrum disorder, obvious dyslexia and tic disorders.
3. Psychoactive substance use or abuse/dependence before depression onset.
4. Obvious active physical diseases.

The inclusion criteria for healthy controls are as follows:

1. No current or previous mental disorder according to DSM-5.
2. Aged 9–18 years.
3. Voluntary participation with written informed consent from the legal guardian.

The exclusion criteria for healthy controls are as follows:

1. Family history of mental disorders.
2. Neurological diseases.
3. Any kind of psychoactive substance use or self-injury.
4. Obvious active physical disease.

### Data collection

#### Demographic information

Demographic information is collected by trained professionals using self-report tools at all time points, excluding T4, which is conducted through a video or telephone follow-up. Demographic information includes birth date, age, gender and education (including parents' educational information).

#### Clinical profile

We compiled a multidimensional clinical profile, including clinical presentation, tobacco/alcohol and substance use, physical health comorbidities, family history and treatment-related information. The constructs of these dimensions are shown in table 1.

**Table 1:** Multidomains of clinical profile, constructs and assessments

Domains	Constructs	Assessments
Clinical presentation	Mental disorder diagnoses	The DSM-5 matched Schedule for Affective Disorders and Schizophrenia for School-Age Children (K-SADS)
	Severity of symptom clusters	
	Severity of depressive symptoms	17-item Hamilton Depression Rating Scale (HAMD-17)
		Montgomery-Åsberg Depression Rating Scale (MADRS)
	Severity of anxiety symptoms	The Hamilton Anxiety Scale (HAMA)
	Severity of hypomanic/manic symptoms	Young Mania Rating Scale (YMRS)
	Severity of psychotic symptoms	six-item version of the Positive and Negative Syndrome Scale (PANSS-6)
	High-risk mental states assessment	Bipolar Prodrome Symptom Scale-Full Prospective (BPSS-FP)
		Structured Interview for Prodromal Syndromes (SIPS)
	Sleep quality and circadian rhythm	Pittsburgh Sleep Quality Index (PSQI), Morningness-Eveningness Questionnaire (MEQ)
	Self-injury and suicidal thoughts and behaviours	Ottawa Self-Injury Inventory (OSI), Columbia Suicide Severity Rating Scale (C-SSRS)
Tobacco/alcohol and substance use	Current and previous smoking status	Smoking questionnaire
		Fagerström Test for Nicotine Dependence (FTND)
	Alcohol and substance use in the life time and last 3 months	Self-designed alcohol use and substance use questionnaire
Physical health comorbidities and family history	Previous and current physical illness of patients	Self-made case report form
	Family history of hypertension, diabetes and thyroid disease	
	Family histories of mental illnesses	Family Interview for Genetic Studies (FIGS)
Treatment-related information	Side effects of antidepressants and adherence	Side effects of antidepressants and adherence
	Treatment response	Reduction rate of HAMD-17
		Clinical Global Impressions-Severity of Illness (CGI-S)

#### Clinical presentation

##### Mental disorder diagnoses

Trained psychiatrists identified MDD (according to DSM-5) using the matched Schedule for Affective Disorders and Schizophrenia for School-Age Children—Present and Lifetime (K-SADS-PL). Psychiatric comorbidities or diagnoses were also labelled as lifetime or current conditions at each time point.

##### Severity of symptom clusters

We focus on four common symptom clusters, including depressive, anxious, manic and psychotic symptoms. The severity of depressive symptoms is assessed with the 17-item Hamilton Depression Rating Scale (HAMD-17)^22^ and the Montgomery-Åsberg Depression Rating Scale (MADRS).^23^ The Hamilton Anxiety Scale (HAMA) is composed of 14 items with a total score of ≥7 indicating the presence of anxiety symptoms.^24^ The Young Mania Rating Scale is adopted to assess any hypomanic/manic symptoms.^25^ The six-item version of the Positive And Negative Syndrome Scale (PANSS-6), which has been shown to be sensitive to the effect of antipsychotic medication, is a measure of the severity of psychotic symptoms.^26^ In PANSS-6, there are three items each for the positive and negative subscale (P1 delusion, P2 conceptual disorganisation and P3 hallucinatory behaviour; N1 blunted affect, N4 passive/apathetic social withdrawal, and N6 lack of spontaneity and flow of conversation).

##### High-risk mental states assessment

Subthreshold clinical symptoms or high-risk symptoms have been previously indicated as risk factors for progression to more severe disorders.^27 28^ As such, to better characterise the full spectrum of depression in adolescents, we also focus on prodromal symptoms. In our cohort study, the subthreshold clinical symptoms include subthreshold depressive symptoms, subthreshold manic symptoms and psychotic high-risk states. According to prior studies, the subthreshold depressive symptoms are defined as (1) the participant experienced a minimum of three DSM-5 depressive symptoms, one of which included a depressed mood, but did not meet the full criteria for a major depressive episode, or the depression was otherwise not specified based on insufficient duration or severity; (2) symptoms represented a clear change from normal functioning, endorsed by self and others who know the person well, and (3) no evidence of major impairment.^29^ The subthreshold hypomanic symptoms are defined as (1) the participant experienced a minimum of three DSM-5 hypomanic symptoms but did not meet the full criteria for a hypomanic or manic episode or was otherwise not specified based on insufficient duration or severity; (2) symptoms represented a clear change from normal functioning, endorsed by self and others who know the person well, and (3) no evidence of major impairment. It is worth mentioning that we introduced the Chinese version of the BPSS-FP, a semi-structured assessment of the prodromal symptoms of bipolar disorder used in those adolescents with depression experiencing subclinical hypomanic symptoms.^20^ To our knowledge, this is the first study to introduce this instrument. Additionally, the Hypomania Checklist-32 (HCL-32) is used to self-assess hypomanic symptoms.^27^ Furthermore, the psychotic high-risk state also was surveyed as outlined in the Structured Interview for Prodromal Syndromes (SIPS).^30^

##### Sleep quality and circadian rhythm

The Pittsburgh Sleep Quality Index (PSQI) is a self-rating instrument to assess sleep quality over the previous 1 month, measuring the contents of subjective sleep quality, sleep latency, sleep duration, sleep efficiency, sleep disturbances, use of sedative-hypnotic drugs and daytime dysfunction.^31^ The Morningness-Eveningness Questionnaire (MEQ) characterises sleep–wake patterns and consists of 19 multiple choice items. According to the total score, the questionnaire categorises individuals into definite morning types and evening types.^32^

##### Self-injury and suicidal thoughts and behaviours

The Ottawa Self-Injury Inventory (OSI) is a self-report measure that assesses the functions and potential addictive features of non-suicidal self-injury (NSSI). We are adopting the Chinese version of OSI to measure the occurrence, frequency and the potential addictive features of self-injury.^33^

The Columbia Suicide Severity Rating Scale (C-SSRS) is a recognised scale that can quantify the severity of suicidal thoughts and suicidal behaviours, including the severity of suicidal thoughts, intensity of suicidal thoughts, suicidal behaviour subscale and lethality subscale.^34^

#### Tobacco/alcohol and substance use

Each time point in which tobacco, alcohol or any kind of psychoactive substance are recorded. Regarding tobacco use, we are adopting a smoking questionnaire focusing on current and previous smoking status. For individuals who smoke regularly, we are using the Fagerström Test for Nicotine Dependence (FTND) to assess tobacco dependence.^35^ A self-designed alcohol use and substance use questionnaire is adopted for their current and lifetime alcohol and substance use.

#### Physical health comorbidities and family history

Any previous and current physical illness of patients is recorded, as well as a family history that includes three diseases: hypertension, diabetes and thyroid disease;^36^this information is prepared for further genetic and epigenetic analysis. In addition, a family history of mental illnesses in first-, second- and third-degree relatives was ascertained by the Family Interview for Genetic Studies (FIGS).^37^

#### Treatment-related information

Information about patient treatment is recorded, including specific drugs, type of treatment, duration, number of hospitalizations, psychotherapy and physical therapy. In addition, information on the side effects of antidepressants and adherence to medication are also investigated. Treatment response has been performed for individuals who were first treated with the antidepressant fluoxetine or escitalopram within 16 weeks by the continuous change of the HAMD-17. A reduction rate of HAMD-17 above 50% from baseline to 8 weeks (T3) is defined as a response, while HAMD-17 total score ≤7 is defined as remission. The Clinical Global Impressions-Severity of Illness (CGI-S) ≤2 is also defined as remission.

### Social and occupational functioning

A functional assessment using the Children’s Global Assessment Scale (CGAS) is assessed at each time point. To avoid confounding the rating with clinical symptoms, we adopted the Social and Occupational Functioning Assessment Scale (SOFAS) for functional assessment.^29^ Moreover, the Functioning Assessment Short Test (FAST) is used for self-reported occupational functioning.^38^

### Environmental risk factors

The original Adolescent Self Rating Life Events Check (ASLEC) List includes 27 life events occurring over the last 6 months.^39^ With the purpose of quantifying persistent negative stress, we have expanded the occurrence of life events to the previous year. The Childhood Trauma Questionnaire (CTQ) is a recognised assessment to investigate different forms of childhood trauma before 16 years of age.^40^ Additionally, we are surveying family environmental factors, such as the number of family members, family income, marital status of parents, including divorce or separation. Additionally, individual risk factors for depression are evaluated, such as neurodevelopmental factors during the mother's pregnancy and perinatal period.

### Objective measures

In our cohort, multilevel units of objective biological indicators are investigated, including neurocognitive function, plasma biomarkers, intestinal flora, anthropometric measurements (eg, height and weight), resting-state electroencephalography (EEG), and structural and functional magnetic resonance imagings (MRIs). All biological sample collections are performed after informed consent and within 3 days of clinical evaluation. Detailed assessment tools and metrics are summarised in table 2.

**Table 2:** Standard assessments and detailed assessments of objective measures

Objective measures	Standard assessments	Detailed assessments
Neurocognitive function	MATRICS Consensus Cognitive Battery (MCCB)	
	Information processing speed	Trail Making Test Part A
		Brief Assessment of Cognition in Schizophrenia: Symbol Coding (BACS-SC)
		Category fluency
	Verbal learning	Hopkins Verbal Learning Test (HVLT)
	Visual learning	Brief Visuospatial Memory Test-Revised (BVMT-R)
	Working memory	Wechsler Memory Scale Spatial span
Anthropometric measurement	Anthropometric measurement	Height
		Weight
		Waist circumference
		Body mass index
		Upper arm circumference
		Blood pressure
Plasma	Metabolic and immune markers	Blood pathology analysis
		Full blood count
		Urea, triglycerides, cholesterol, apolipoprotein
		Thyroid function
		Fasting blood glucose, insulin, β-hydroxybutyric acid
	More extensive molecular biologic marker screening	DNA, RNA, neuroimmune cytokines, protein, exosomes, etc
Faeces	Intestinal flora	Abundance of microbial compositions, Alpha-diversity
EEG	Resting-state EEG	Delta, Theta, Alpha, Beta and Gamma bands
Brain structure and function	Brain structure	Cortical thickness, surface area, cortical and subcortical volume and mean curvature
	Brain function	Metrics of local brain activity: ALFF, ReHo, DC, VMHC, etc
		Functional connectivity: static and dynamic FC
		Global and Network Metrics: small world, global efficiency, rich-club, clustering coefficient, shortest path length, betweenness-centrality local efficiency, etc

ALFF, amplitude of low-frequency fluctuation; DC, degree centrality; EEG, electroencephalography; FC, functional connectivity; MATRICS, Measurement and Treatment Research to Improve Cognition in Schizophrenia; ReHo, regional homogeneity; VMHC, voxel-mirrored homotopic connectivity.

### Neurocognitive function

In our cohort study, neurocognitive function is characterised by using the Chinese version of the Measurement and Treatment Research to Improve Cognition in Schizophrenia (MATRICS) Consensus Cognitive Battery (MCCB), which has demonstrated clinical validity and test–retest reliability in patients with MDD.^41^ Taking into account the uniqueness of the adolescent population and time constraints, we choose assessment of four domains of information processing speed, verbal learning, visual learning, and working memory, which consisting of six items of Trail Making Test Part A, Brief Assessment of Cognition in Schizophrenia: Symbol Coding (BACS-SC), Hopkins Verbal Learning Test (HVLT), Brief Visuospatial Memory Test-Revised (BVMT-R), Wechsler Memory Scale Spatial span and Category Fluency.

### Biological sample collection

Blood samples are separated into plasma and red blood cells (for DNA isolation). Whole blood is also collected in Tempus tubes for direct isolation of RNA. Faeces from participants are partly collected for intestinal flora analysis. Brain electrical activity and brain MRI are also recorded.

### Resting-state electroencephalography

The EEG data are collected through a 30-channel EEG cap (LT 37) following the extended 10–20 system, and the right mastoid is taken as a signal reference. The sampling rate is set to 250 Hz. We are adopting a 40-channel amplifier NuAmps (Compumedics; Neuroscan, Australia) to collect EEG signals for part of the participants. During data collection, all electrode impedances are kept below 5kΩ to ensure signal quality.

### Brain structure and function

MRI scans are performed within 3 days of clinical assessment and biological sample collections. All adolescents are scanned for structural and functional MRI data on a 3.0 Tesla MRI system. Foam pads and headphones are used to minimise head motion and scanner noise. High-resolution T1-weighted images are collected using a fast gradient echo sequence with the following parameters: repetition time (TR)/echo time (TE)=8.2 ms/3.8 ms, field of view (FOV)=256×256 mm^2^, matrix=256×256, sagittal slices=188, thickness=1 mm, and voxel size=1×1×1 mm^3^. Functional MRI data are acquired using a gradient-echo echo-planar imaging sequence with the following parameters: TR=2000 ms, TE=30 ms, flip angle (FA)=90 degrees, FOV=220×220 mm^2^, matrix=64×64, axial slices=33, thickness=4 mm, gap=0.6 mm and voxel size=3.44×3.44×4 mm^3^.

### Follow-up

The patient group is invited to complete all the follow-up assessments. We planned several ways to improve the follow-up rate. First, our team has sufficient human resources, including two full-time research psychiatrists, three research assistants, two graduate students and a specialist for project management. Both researchers and clinical psychiatrists fully collaborate, promoting a successful follow-up. Second, the division of labour is clear. A specialist is responsible for the follow-up contact and fixed, accountable assistants are responsible for the follow-up reception and scheduling of each of the examinationss, which guarantees good communication between our team and the adolescents' families. Third, we fully inform participants and their families to ensure they understand the programme's purpose and benefits at baseline enrolment; this could be further elaborated in follow-up sessions. Fourth, we provide practical help to patients (eg, offering ways for recording their mood changes throughout the day, training for early recognition of hypomania, assisting with doctors’ appointments when necessary, and giving psychological advice when needed). Fifth, we invite parents into an exclusive WeChat group after the enrolment and send them articles about mood disorders to improve the families' awareness of the disorders.

**Figure 3:**
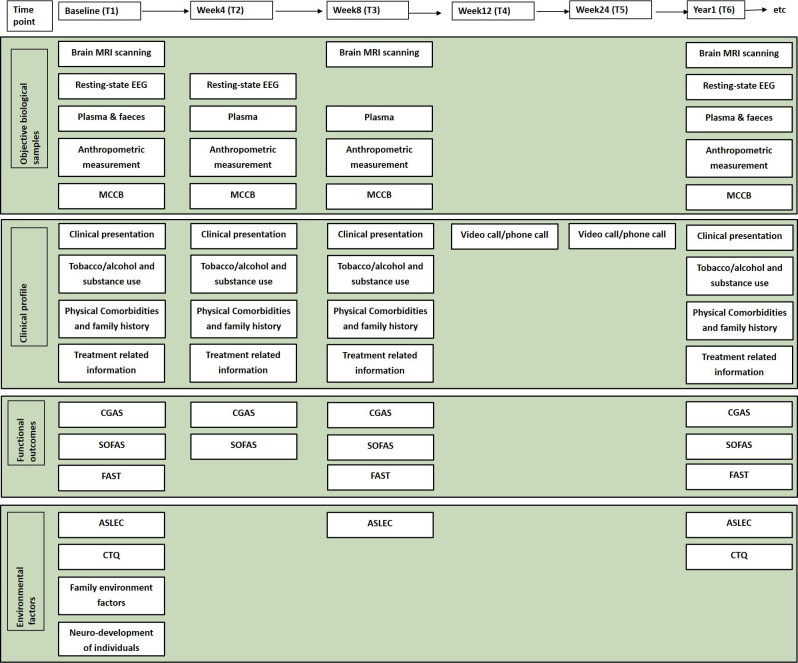
Multidimensional assessment and specific follow-up times. ASLEC, Adolescent Self Rating Life Events Check; CGAS, Children's Global Assessment Scale; CTQ, Childhood Trauma Questionnaire; EEG, electroencephalography; FAST, Functioning Assessment Short Test; MCCB, Measurement and Treatment Research to Improve Cognition In Schizophrenia (MATRICS) Consensus Cognitive Battery; MRI, magnetic resonance imaging; SOFAS, Social and Occupational Functioning Assessment Scale.

Specific follow-up times and the content of the multidimensional framework assessment are summarised in figure 3.

### Statistical analysis

All data are collected by Epidata3.0. Data analysis will be done using the Statistical Package for Social Sciences 22 (SPSS 22). Statistical significance will be set at two-sided p<0.05 for behavioural and demographic data. Group differences in demographic, clinical, cognitive function data and cortical thickness will be assessed via analysis of variance or χ^2^ tests where relevant. The data of pretreatment/post-treatment will be analysed by the paired sample t-test. We will use multinomial models to analyse drug predictive associations with adolescent depression. For the trajectory of clinical symptoms analysis, according to previous studies, we will use a novel form of growth mixture modelling with structured residuals (GMM-SR) in MPlus (V.8.0) to classify youths into distinct subgroups.^42^

### Strengths and limitations of this study

The current study addresses the lack of longitudinal cohort studies with large depression samples in Chinese adolescents. It will provide information on the trajectory of clinical symptoms in Chinese adolescents in the real world. Furthermore, it will track multidimensional outcomes, including circadian rhythm, physical comorbidity, tobacco/alcohol and substance use, self-injury and suicide, which can provide rich information to paediatric psychiatrists for early intervention. Most importantly, we launched a comprehensive framework with a long-term longitudinal study, collecting multiple units of objective markers at different time points. This framework fully considers the complex clinical heterogeneity of adolescent depression, facilitating the identification of biomarkers reflecting relationships among genetic, molecular neural circuitry levels and behavioural-level abnormalities. Our study will inform the development of early interventions in adolescents with depression and reduce the burden of the disease. The disadvantage of this comprehensive study is that acquisition of clinical information will occur over a long period, which may lead to an increased rate of subject loss. We will take measures, such as step-by-step evaluation and strengthening health education, to encourage patient follow-up.

## Data Availability

There are no data in this work.

## References

[1] National Institute of Mental Health. Major depression. Available: https://www.nimh.nih.gov/health/statistics/major-depression [Accessed 13 Dec 2018].

[2] Weinberger AH, Gbedemah M, Martinez AM, et al. Trends in depression prevalence in the USA from 2005 to 2015: widening disparities in vulnerable groups. Psychol Med 2018;48:1308–15. 10.1017/S0033291717002781 29021005

[3] Avenevoli S, Swendsen J, He J-P, et al. Major depression in the national comorbidity survey-adolescent supplement: prevalence, correlates, and treatment. J Am Acad Child Adolesc Psychiatry 2015;54:37–44. 10.1016/j.jaac.2014.10.010 25524788 PMC4408277

[4] Li F, Cui Y, Li Y, et al. Prevalence of mental disorders in school children and adolescents in China: diagnostic data from detailed clinical assessments of 17,524 individuals. J Child Psychol Psychiatry 2022;63:34–46. 10.1111/jcpp.13445 34019305

[5] Kwong ASF, López-López JA, Hammerton G, et al. Genetic and environmental risk factors associated with trajectories of depression symptoms from adolescence to young adulthood. JAMA Netw Open 2019;2:e196587. 10.1001/jamanetworkopen.2019.6587 31251383 PMC6604106

[6] Que J, Lu L, Shi L. Development and challenges of mental health in China. Gen Psychiatr 2019;32:e100053. 10.1136/gpsych-2019-100053 31179426 PMC6551437

[7] Thapar A, Collishaw S, Pine DS, et al. Depression in adolescence. Lancet 2012;379:1056–67. 10.1016/S0140-6736(11)60871-4 22305766 PMC3488279

[8] Yang B, Wei J, Ju P, et al. Effects of regulating intestinal microbiota on anxiety symptoms: a systematic review. Gen Psychiatr 2019;32:e100056. 10.1136/gpsych-2019-100056 31179435 PMC6551444

[9] Selph SS, McDonagh MS. Depression in children and adolescents: evaluation and treatment. Am Fam Physician 2019;100:609–17. 31730312

[10] Geller B, Tillman R, Craney JL, et al. Four-year prospective outcome and natural history of mania in children with a prepubertal and early adolescent bipolar disorder phenotype. Arch Gen Psychiatry 2004;61:459–67. 10.1001/archpsyc.61.5.459 15123490

[11] Judd LL, Schettler PJ, Akiskal H, et al. Prevalence and clinical significance of subsyndromal manic symptoms, including irritability and psychomotor agitation, during bipolar major depressive episodes. J Affect Disord 2012;138:440–8. 10.1016/j.jad.2011.12.046 22314261 PMC3677770

[12] Angst J, Cui L, Swendsen J, et al. Major depressive disorder with subthreshold bipolarity in the National Comorbidity Survey Replication. Am J Psychiatry 2010;167:1194–201. 10.1176/appi.ajp.2010.09071011 20713498 PMC3145248

[13] Rao U, Ryan ND, Birmaher B, et al. Unipolar depression in adolescents: clinical outcome in adulthood. J Am Acad Child Adolesc Psychiatry 1995;34:566–78. 10.1097/00004583-199505000-00009 7775352

[14] Cardoso de Almeida JR, Phillips ML. Distinguishing between unipolar depression and bipolar depression: current and future clinical and neuroimaging perspectives. Biol Psychiatry 2013;73:111–8. 10.1016/j.biopsych.2012.06.010 22784485 PMC3494754

[15] Patton GC, Coffey C, Romaniuk H, et al. The prognosis of common mental disorders in adolescents: a 14-year prospective cohort study. Lancet 2014;383:1404–11. 10.1016/S0140-6736(13)62116-9 24439298

[16] Metzak PD, Addington J, Hassel S, et al. Functional imaging in youth at risk for transdiagnostic serious mental illness: initial results from the PROCAN study. Early Interv Psychiatry 2021;15:1276–91. 10.1111/eip.13078 33295151

[17] Strawn JR, Mills JA, Croarkin PE. Switching selective serotonin reuptake inhibitors in adolescents with selective serotonin reuptake inhibitor-resistant major depressive disorder: balancing tolerability and efficacy. J Child Adolesc Psychopharmacol 2019;29:250–5. 10.1089/cap.2018.0145 30810350 PMC6534091

[18] Carpenter JS, Iorfino F, Cross S, et al. Cohort profile: the Brain and Mind Centre Optymise cohort: tracking multidimensional outcomes in young people presenting for mental healthcare. BMJ Open 2020;10:e030985. 10.1136/bmjopen-2019-030985 PMC7170572 32229519

[19] Li C-T, Bai Y-M, Huang Y-L, et al. Association between antidepressant resistance in unipolar depression and subsequent bipolar disorder: cohort study. Br J Psychiatry 2012;200:45–51. 10.1192/bjp.bp.110.086983 22016435

[20] Correll CU, Olvet DM, Auther AM, et al. The Bipolar Prodrome Symptom Interview and Scale-Prospective (BPSS-P): description and validation in a psychiatric sample and healthy controls. Bipolar Disord 2014;16:505–22. 10.1111/bdi.12209 24807784 PMC4160534

[21] American Psychiatric Association. Diagnostic and statistical manual of mental disorders. Washington, DC: American Psychiatric Association, 1980.

[22] Hamilton M. The Hamilton rating scale for depression. In: Assessment of depression. Springer, 1986: 143–52.

[23] Montgomery SA, Åsberg M. A new depression scale designed to be sensitive to change. Br J Psychiatry 1979;134:382–9. 10.1192/bjp.134.4.382 444788

[24] Maier W, Buller R, Philipp M, et al. The Hamilton anxiety scale: reliability, validity and sensitivity to change in anxiety and depressive disorders. J Affect Disord 1988;14:61–8. 10.1016/0165-0327(88)90072-9 2963053

[25] Young RC, Biggs JT, Ziegler VE, et al. A rating scale for mania: reliability, validity and sensitivity. Br J Psychiatry 1978;133:429–35. 10.1192/bjp.133.5.429 728692

[26] Østergaard SD, Lemming OM, Mors O, et al. PANSS-6: a brief rating scale for the measurement of severity in schizophrenia. Acta Psychiatr Scand 2016;133:436–44. 10.1111/acps.12526 26558537

[27] Hauser M, Correll CU. The significance of at-risk or prodromal symptoms for bipolar I disorder in children and adolescents. Can J Psychiatry 2013;58:22–31. 10.1177/070674371305800106 23327753 PMC4010197

[28] Kelleher I, Cannon M. Psychotic-like experiences in the general population: characterizing a high-risk group for psychosis. Psychol Med 2011;41:1–6. 10.1017/S0033291710001005 20624328

[29] Samara MT, Engel RR, Millier A, et al. Equipercentile linking of scales measuring functioning and symptoms: examining the GAF, SOFAS, CGI-S, and PANSS. Eur Neuropsychopharmacol 2014;24:1767–72. 10.1016/j.euroneuro.2014.08.009 25219937

[30] Miller TJ, McGlashan TH, Rosen JL, et al. Prodromal assessment with the structured interview for prodromal syndromes and the scale of prodromal symptoms: predictive validity, interrater reliability, and training to reliability. Schizophr Bull 2003;29:703–15. 10.1093/oxfordjournals.schbul.a007040 14989408

[31] Buysse DJ, Reynolds CF, Monk TH, et al. The Pittsburgh sleep quality index: a new instrument for psychiatric practice and research. Psychiatry Res 1989;28:193–213. 10.1016/0165-1781(89)90047-4 2748771

[32] Horne JA, Ostberg O. A self-assessment questionnaire to determine morningness-eveningness in human circadian rhythms. Int J Chronobiol 1976;4:97–110. 1027738

[33] Zhang F, Cheng WH, Xiao ZP. Study on reliability and validity of Chinese version of Ottawa self-injury inventory. Journal of Shanghai Jiaotong University 2015;35:460–4.

[34] Posner K, Brown GK, Stanley B, et al. The Columbia-Suicide Severity Rating Scale: initial validity and internal consistency findings from three multisite studies with adolescents and adults. Am J Psychiatry 2011;168:1266–77. 10.1176/appi.ajp.2011.10111704 22193671 PMC3893686

[35] Fagerstrom KO, Schneider NG. Measuring nicotine dependence: a review of the Fagerstrom tolerance questionnaire. J Behav Med 1989;12:159–82. 10.1007/BF00846549 2668531

[36] Chaturvedi SK, Manche Gowda S, Ahmed HU, et al. More anxious than depressed: prevalence and correlates in a 15-nation study of anxiety disorders in people with type 2 diabetes mellitus. Gen Psychiatr 2019;32:e100076. 10.1136/gpsych-2019-100076 31552386 PMC6738670

[37] Initiative NG. Family interview for genetic studies (figs): manual for figs. National Institute of Mental Health, 1992.

[38] Zhang Y, Long X, Ma X, et al. Psychometric properties of the Chinese version of the functioning assessment short test (fast) in bipolar disorder. J Affect Disord 2018;238:156–60. 10.1016/j.jad.2018.05.019 29883937

[39] Chen H, Jia CX, Liu XC. Psychometric properties and application of Adolescent Self-Rating Life Events Checklist(ASLEC). Chinese Journal of Public Health 2016;32:1116–9.

[40] Bernstein DP, Fink L, Handelsman L, et al. Initial reliability and validity of a new retrospective measure of child abuse and neglect. Am J Psychiatry 1994;151:1132–6. 10.1176/ajp.151.8.1132 8037246

[41] Liang S, Xing X, Wang M, et al. The MATRICS consensus cognitive battery: psychometric properties of the Chinese version in young patients with major depression disorder. Front Psychiatry 2021;12:745486. 10.3389/fpsyt.2021.745486 34777049 PMC8580868

[42] Hawrilenko M, Masyn KE, Cerutti JK. Individual differences in the course of youth depression: the importance of renitence and reversion. medRxiv - Psychiatry and Clinical Psychology [Preprint]. May 20, 2020. 10.1101/19012872

